# Coordination of a Dirhodium(II) Center to Methionine and Cysteine Side Chains: Evidence from X-Ray Structure of the Adduct Formed by Dirhodium Tetraacetate with a C-Phycocyanin

**DOI:** 10.3390/ijms262311492

**Published:** 2025-11-27

**Authors:** Giarita Ferraro, Paola Imbimbo, Romualdo Troisi, Daria Maria Monti, Antonello Merlino

**Affiliations:** Department of Chemical Sciences, University of Naples Federico II, Via Cintia, 80126 Napoli, Italy; giarita.ferraro@unina.it (G.F.); paola.imbimbo@unina.it (P.I.); romualdo.troisi@unina.it (R.T.); mdmonti@unina.it (D.M.M.)

**Keywords:** dirhodium compounds, paddlewheel dimetallic complexes, metal-protein adducts, metallodrugs, protein metalation

## Abstract

Upon reaction of dirhodium tetraacetate ([Rh_2_(μ-O_2_CCH_3_)_4_]) and some [Rh_2_(μ-O_2_CCH_3_)_4_] derivatives with proteins, dimeric Rh-Rh units (diRh) or monometallic moieties can bind the side chains of His, Cys, Met, Asp, Asn, Arg and Lys, and the C-terminal carboxylate. However, structural data on the interaction between the diRh center and Cys and Met side chains within the protein environment are still missing. Here, we report the X-ray structure of the adduct that [Rh_2_(μ-O_2_CCH_3_)_4_] forms with C-phycocyanin from *Galdiera phlegrea* at 2.17 Å resolution. Twelve diRh binding sites were found on the protein structure, two for each (αβ) unit. Dimetallic fragments were observed close to the side chains of Met30 of β-chains and of Cys73 of α-chains. To the best of our knowledge, the results provide the first unambiguous crystallographic observation of the diRh center binding to Met and Cys protein residues. DiRh binding does not alter overall protein structure and stability. This result will help in the design of new dirhodium-based artificial metalloenzymes.

## 1. Introduction

Paddlewheel dirhodium(II) complexes of general formula [Rh_2_(O_2_CR)_4_]L_2_ (R=CH_3_–, CH_3_CH_2_–, etc.) contain two Rh atoms in the oxidation state +2, held together by a single metal-to-metal bond. In these structures, the dimetallic center is generally surrounded by four equatorial O_2_CR ligands and two axial L ligands along the Rh-Rh axis [1,2]. These complexes have been extensively studied as catalysts for several reactions [3,4,5,6,7,8,9], including photochemical hydrogen evolution [10], selective intermolecular C–H and S-H functionalization [11,12], synthesis with α-diazocarbonyl compounds [13,14], and selective olefin cyclopropanation [15,16]. These molecules can also be used as detectors for ammonia and nitric oxide [17,18].

**Figure 1:**
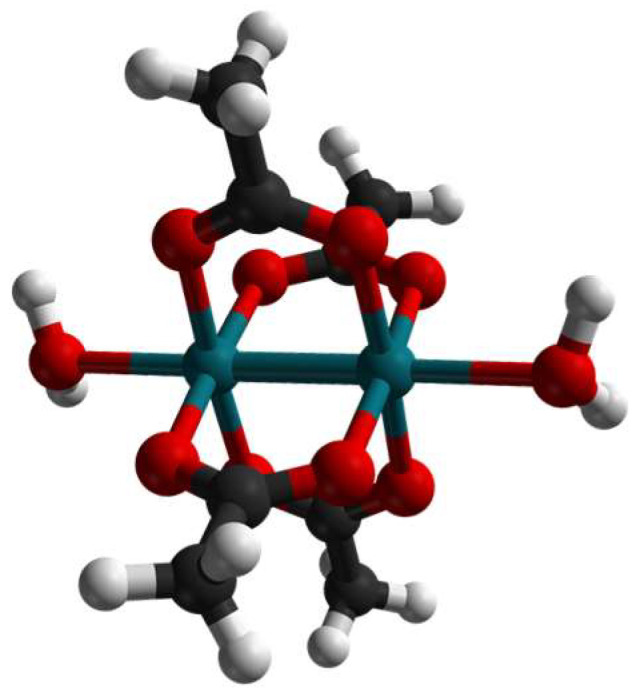
Paddlewheel structure of [Rh_2_(OAc)_4_]. Two water molecules are the axial ligands. Rh atoms are colored in deep teal, C atoms are depicted in black, O atoms in red, and H atoms in white (CSD entry: ACAQRH10) [30].

It has also been shown that dirhodium (diRh) compounds can act as anticancer and antibacterial agents [19,20,21,22]; as an example, dirhodium tetraacetate ([Rh_2_(µ-O_2_CCH_3_)_4_] or [Rh_2_(OAc)_4_], Figure 1) is able to treat Ehrlich ascites in model mice, L1210 tumors, sarcoma 180, and P388 leukemia [19,20,21].

Due to their potential anticancer activities [22], dirhodium compounds have been extensively investigated for their interaction with nucleic acids [23]. Dirhodium(II) carboxylates bind nucleotides [24], single- and double-stranded DNA [25], forming interfilamentous cross-links [26,27] that interfere with DNA replication and transcription [23]. It has been shown that interligand interactions affect the binding properties of these compounds to nucleobases [27,28,29].

A detailed definition of the binding of the diRh core to DNA has been recently obtained by combining X-ray crystallography and mass spectrometry (MS) data. We have demonstrated that the diRh center binds adenines of DNA via axial coordination [31]. Thus, diRh compounds react with DNA differently from cisplatin and its derivatives, which prefer guanines [32,33].

The interaction of diRh complexes with amino acids [16], peptides [34], and proteins [35] has also been extensively investigated. For example, Rh K-edge extended X-ray absorption fine structure (EXAFS), UV-Vis absorption spectroscopy, and electrospray ionization MS (ESI-MS) have demonstrated that [Rh_2_(OAc)_4_] binds Met at the axial site and that two Met groups can also replace the equatorial acetate ligands [36,37]. Spectroscopic and MS data have also shown that [Rh_2_(OAc)_4_] binds the β-domain of β-metallothionein Rh1a with the dimetallic core that is retained and the acetate ligands that are replaced by Cys SG atoms [38]. Time-dependent MS experiments revealed that the metal binding depends on the environmental conditions: at neutral pH, one, two, or three diRh centers bind β-metallothionein Rh1a, losing the equatorial ligands [39,40], while at pH < 2, up to six [Rh_2_(OAc)_4_] units bind β-metallothionein Rh1a [40]. The in-solution reactivity of [Rh_2_(OAc)_4_] and [Rh_2_(OAc)_2_(bpy)_2_] (bpy = bipyridine) with Human Serum Albumin (HSA), the most abundant plasma protein, showed that the metal complexes bind the protein with a molar ratio of 8:1 and 7:1, respectively [41,42]. Binding of [Rh_2_(OAc)_4_] and [Rh_2_(OAc)_2_(bpy)_2_] to HSA reduces the protein α-helix content. X-ray absorption spectroscopy analysis then revealed that [Rh_2_(OAc)_4_] binds the imidazole of His or the SG atom of the free Cys34 [42]. DiRh complexes can also act as protein cross-linking agents [43].

We solved the X-ray structures of the reaction products of [Rh_2_(OAc)_4_], *cis*-[Rh_2_(μ-O_2_CCF_3_)_2_(OAc)_2_], and [Rh_2_(μ-O_2_CCF_3_)_3_(OAc)] with the model proteins hen egg white lysozyme (HEWL) [44,45,46,47] and bovine pancreatic ribonuclease (RNase A) [48,49], demonstrating the binding of the diRh center to His side chains at the axial site and to Asp, Asn, and Lys side chains at equatorial sites, the binding of monometallic fragments to Lys, Asn, His/Arg, and that of a Rh(III)-O-Rh(III) structural motif to the C-terminal tail at both cryogenic and physiological temperatures.

Notably, axial binding of the protein residue side chain to the diRh core significantly alters the reactivity of the metal compounds. Indeed, in the reaction of the diRh/RNase A adduct with imidazole in the solid state, an unexpected reaction product was obtained [50]. We have also shown that cross-linked crystals of diRh/RNase A adducts can act as catalysts for the olefin cyclopropanation reaction and self-coupling of diazo compounds [49].

In our continuous effort to study the interaction of paddlewheel dirhodium complexes and biological macromolecules [51] and to obtain new crystals of metal/protein adducts that could be used as heterogenous catalysts, we have looked for new protein crystals that could be used to immobilize the diRh center. Since C-phycocyanins have been used as model systems for crystallization studies [52], during this search, we have treated crystals of C-phycocyanin from *Galdiera phlegrea* (GpPC), available in our laboratory [53,54], with [Rh_2_(OAc)_4_]. Here, we report the X-ray structure of the adduct that [Rh_2_(OAc)_4_] forms with GpPC (diRh/GpPC adduct). The structure shows the first crystallographic observation of the binding of the dirhodium core to Met and Cys side chains. Furthermore, we compared the thermal stability of the adduct with that of the metal-free protein. Results indicate that diRh binding to GpPC does not alter its overall structure and stability.

## 2. Results and Discussion

### 2.1. Structure of the diRh/GpPC Adduct

The structure of diRh/GpPC was solved at 2.17 Å resolution using data collected at Diamond Light Source, Oxfordshire, United Kingdom, on GpPC crystals, grown as described in ref. [53] and treated with a saturated solution of dirhodium tetraacetate for 5 days. Data collection and refinement statistics are reported in Table 1.

**Figure 2:**
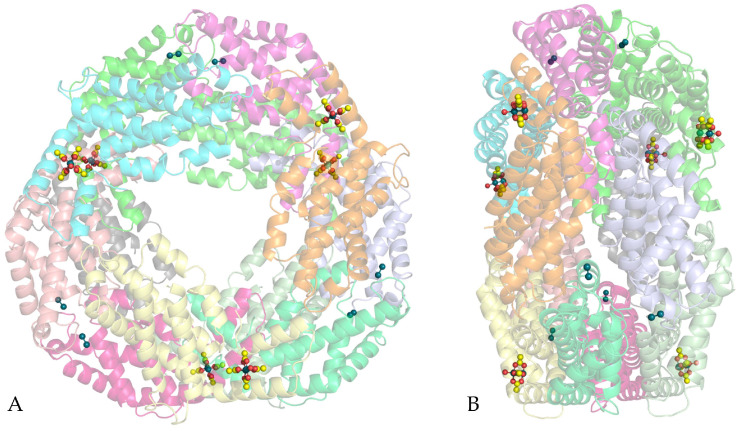
Overall structure of the diRh/GpPC adduct. Frontal view in panel (**A**) and lateral view in panel (**B**). DiRh-containing fragments are highlighted in ball-and-stick (C atoms in yellow, O atoms in red, Rh atoms in deep teal).

**Figure 3:**
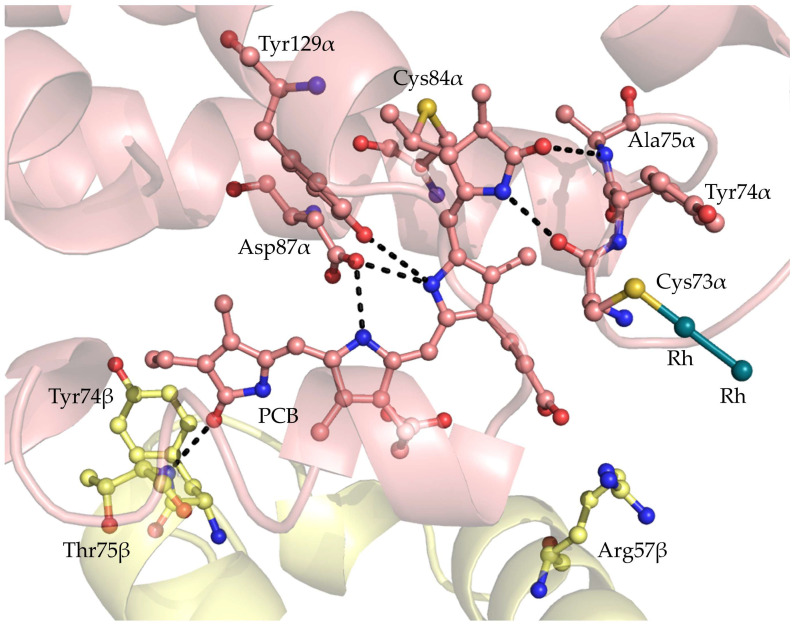
PCB interaction in the α-chain (pink). PCBα forms hydrogen bonds (black dashed lines) with the side chains of Asp87α and Tyr129α, the main chain atoms of residue Ala75α and Cys73α, whose side chain binds a dirhodium center, and with Tyr74, Thr75, and Arg57 from the β-chain (yellow).

The structure of the adduct, reported in Figure 2, shows the typical [(αβ)_3_]_2_ assembly of GpPC. The main structural features of GpPC in the adduct, including methylation of the ND2 atom of Asn72β, are very similar to those of the metal-free GpPC [53]. Root mean square deviations (r.m.s.d.) of the carbon alpha atoms between these structures are as low as 0.36 Å. Dirhodium centers were observed on the protein surface (Figure 2), bound to the side chains of Cys73 of the α-chain and of Met30 of the β-chain in all six αβ GpPC units. The presence of Rh centers at these sites is confirmed by inspection of anomalous difference electron density (e.d) maps. Thus, the whole GpPC structure contains twelve diRh-containing fragments. In the adduct, GpPC remains able to bind the phycocyanobilin (PCB) chromophore, whose conformation and interaction with protein residues are not affected by the dirhodium compound binding, although in the α-chains, the diRh binding site is not far from PCB (Figure 3).

**Figure 4:**
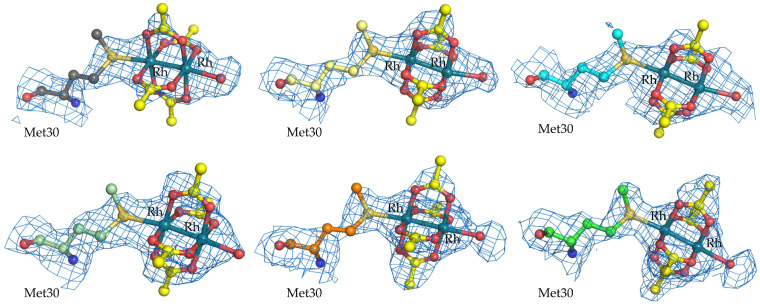
DiRh binding sites close to the side chains of Met30 in the six β-chains of diRh/GpPC. 2Fo-Fc e.d. maps are contoured at 1.0 σ (blue).

The dirhodium centers bound to Met30 of the β-chains are rather well defined in the Fourier difference e.d. maps (Figure 4). The dimetallic core retains the four acetate ligands at the equatorial sites, with a water molecule at the axial site completing the diRh coordination sphere (Figure 4). Geometric parameters of the six [Rh_2_(OAc)_4_] bound to Met30β side chains of GpPC are reported in Table 2. At these sites, one Rh atom coordinates the SD atom of the Met at the axial site, with an average distance equal to 2.46 ± 0.05 Å. This bond length is slightly lower than expected, since the range of Rh-S bond lengths observed in small molecules and expected for SD-diRh coordination is within 2.52 and 2.56 Å [36,55]. The Rh-Rh distance is 2.38 ± 0.01 Å, in good agreement with the expected value (2.39 Å, [55]). The occupancy of Rh atoms at the six binding sites is almost identical, with a value of 0.60 in chain L and 0.50 in the other chains. B-factors are within the range from 24.0 to 59.7 Å^2^.

**Figure 5:**
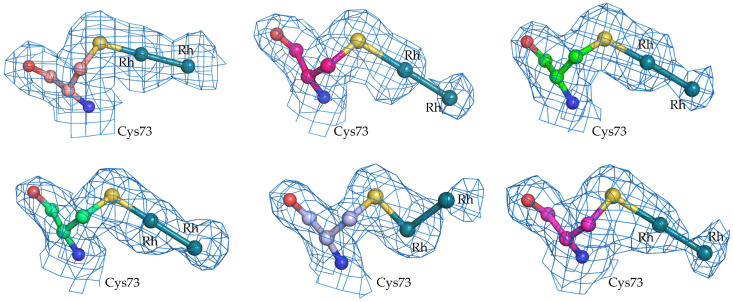
DiRh binding sites close to the side chains of Cys73 in the six α-chains of diRh/GpPC. 2Fo-Fc e.d. maps are contoured at 1.0 σ (blue).

The e.d. maps of diRh centers at the level of Cys73 are less well defined, likely due to the low occupancies of the dimetallic centers close to this site. At these sites, equatorial ligands were not modeled (Figure 5). Dirhodium centers are bound to the SG atom of the Cys at the axial site, with the only exception being Cys73 of chain I, where dirhodium appears equatorially coordinated. Geometric parameters of the diRh-containing fragments bound to Cys73α side chains of GpPC are reported in Table 2. Rh-Rh distance is 2.40 ± 0.01 Å, SG-Rh distances are on average at 2.26 ± 0.17 Å, in line with DFT calculations [55]. Occupancy of Rh atoms is within the range of 0.20–0.40 and B-factors within the range of 30.9–65.7 Å^2^_._

### 2.2. Comparison with Literature Data

In total, 23 examples of Rh/protein structures with diRh centers bound to protein atoms are reported in the Protein Data Bank (Tables S1–S3). These structures have been obtained upon reaction of [Rh_2_(OAc)_4_], [Rh_2_(μ-O_2_CCF_3_)_3_(OAc)], and *cis*-[Rh_2_(μ-O_2_CCF_3_)_2_(OAc)_2_] with HEWL [44,45,46,47] and RNase A [48,49], both by soaking and co-crystallization procedures, with similar results.

Data analysis shows that diRh centers bind preferentially to the His side chain at the axial site. The diRh core can also be coordinated by two side chains at the same time, as occurs in the case of Asn93/Lys96 of HEWL [44,45,46,47]. In some cases, diRh complexes can degrade upon reaction with proteins, and monometallic fragments can bind a single residue (Asp or Lys side chains) [44,45,46] or even more than one side chain at the same time, as it occurs in the case of His15/Arg14 of HEWL [44,47]. Finally, Rh centers can oxidize, forming a Rh(III)-O-Rh(III) structural motif that can bind carboxylates, as at the C-terminal tail, also acting as a protein cross-linker [44,47].

Thus, the structure reported here provides the first unambiguous crystallographic observation of diRh binding to methionine and cysteine side chains. Overall, these results demonstrate that the dimetallic center of [Rh_2_(OAc)_4_] can coordinate Met and Cys side chains at the axial site without breakage of the metal–metal bond, previously observed in the case of the β-domain of β-metallothionein Rh1a [40], and without losing acetate ligands, as suggested by reactivity studies of the metal compound with DL-methionine [36,37].

### 2.3. In Solution Secondary Structure and Thermal Stability Analysis of diRh/GpPC Adduct

**Figure 6:**
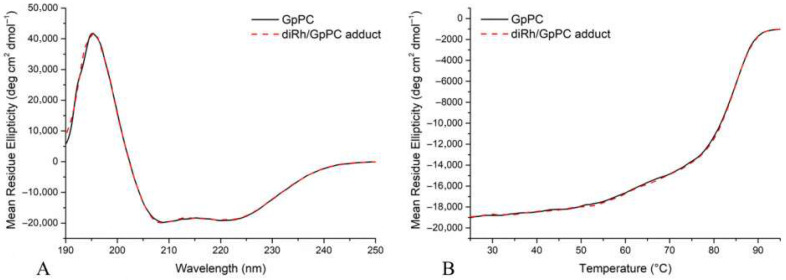
Far-UV CD spectra (**A**) recorded at 25 °C and thermal denaturation profiles (**B**), obtained by monitoring the molar ellipticity at 222 nm as a function of temperature, for GpPC (solid black line) and the diRh/GpPC adduct (dashed red line). Measurements were carried out in 10 mM Tris-HCl, pH 7.0, using a GpPC αβ unit concentration of 0.2 mg mL^−1^.

To characterize the effect of [Rh_2_(OAc)_4_] binding to GpPC in solution from a structural point of view and to monitor the secondary structure content variation upon metal–protein interaction, circular dichroism (CD) spectroscopy was used (Figure 6A,B). Far UV-CD spectra of GpPC in the absence and in the presence of [Rh_2_(OAc)_4_] were recorded. In particular, the protein was incubated for 24 h with dirhodium tetraacetate in a 1:3 protein αβ unit-to-metal molar ratio in 10 mM Tris-HCl, pH 7.0. The superposition of CD spectra of metal-free GpPC and of the diRh/GpPC adduct shows that diRh binding does not affect protein secondary structure organization. Indeed, GpPC displays the typical spectral profile of the α-helix secondary structure with two minima at 208 and 222 nm and a maximum at 195 nm (Figure 6A). In addition, GpPC thermal stability was tested following the CD signal at 222 nm as a function of temperature (25–95 °C) (Figure 6B). The denaturation curves of metal-free GpPC and of the diRh/GpPC adduct are completely superimposable, showing a melting temperature of 85 ± 1 °C under the investigated experimental conditions.

## 3. Materials and Methods

### 3.1. Crystallization of GpPC and Formation of the diRh/GpPC Adduct

[Rh_2_(OAc)_4_] was acquired from Sigma Chemical Co., St. Louis, MO, USA and used without further purification. GpPC was extracted from the algae and purified using the procedure described elsewhere [54].

Crystals of GpPC were obtained using previously reported methods and crystallization conditions of the protein [53]. Briefly, to obtain crystals of the metal-free GpPC, 1 µL of the protein (about 37 mg/mL) was mixed with 1 µL of the reservoir solution containing 0.10–0.20 M magnesium chloride, 0.10 M Hepes pH 6.5, and 9.0–10.0% (*w*/*v*) PEG 4000. Crystals of the diRh/GpPC adduct were obtained by a soaking strategy, i.e., treating metal-free GpPC crystals with a solution of the reservoir saturated with [Rh_2_(OAc)_4_] for 5 days at 20 °C.

diRh/GpPC crystals were then fished with a nylon loop and frozen in liquid nitrogen, once cryoprotected with 20% glycerol.

### 3.2. Data Collection, Structure Solution, and Refinement

X-ray diffraction data were collected at 100 K on the I04 beamline of Diamond Light Source (Diamond House, Harwell Science and Innovation Campus, Oxfordshire, OX11 0DE, UK) and processed using Autoproc [56]. Data collection statistics are reported in Table 2. The phase problem was solved by molecular replacement using Phaser software (v. 2.8.3) [57] and the [(αβ)_3_]_2_ structure of GpPC with Protein Data Bank (PDB) code 6Y3D [53] as a search model. The structure was refined using Refmac5 [58] from CCP4 suite [59]. Electron density maps were manually inspected using Coot [60]. This software was also used for model building, adjustments, optimization, and validation. Figures were generated using PyMol (www.pymol.org). Structure factors and structural coordinates were deposited in the PDB under the accession code 9T05.

### 3.3. Circular Dichroism

Circular dichroism (CD) measurements were performed on a Jasco J-1500 spectropolarimeter (Jasco Corporation, Easton, MD, USA) equipped with a Peltier temperature controller. Far-UV CD spectra were recorded at a GpPC αβ unit concentration of 0.2 mg ml^−1^ in 10 mM Tris-HCl, pH 7.0, at 25 °C, using a cell with an optical path length of 0.1 cm. Spectra were collected at a scanning speed of 50 nm min^−1^, with a response time of 2 s, a data pitch of 0.2 nm, and a bandwidth of 2.0 nm, and were obtained by averaging three consecutive scans. Thermal unfolding profiles were obtained by monitoring the CD signal at 222 nm while heating the samples from 25 to 95 °C at a rate of 1.0 °C min^−1^. The melting temperatures were determined from the first derivative of the melting curves. Experiments on diRh/GpPC adduct were carried out on samples in which a threefold molar excess of [Rh_2_(OAc)_4_] relative to the GpPC αβ unit had been incubated for 24 h at 20 °C prior to analysis. All measurements were performed in triplicate.

## 4. Conclusions

Dirhodium tetracarboxylate binding to proteins has been receiving increasing attention in recent years due to the potential role of diRh/protein adducts in catalysis and biomedical applications [35]. Here, we have analyzed the structure of the adduct formed upon the reaction of C-phycocyanin from *Galdiera phlegrea* [53,54] with [Rh_2_(OAc)_4_] and have studied its thermal stability by circular dichroism. This system was chosen because C-phycocyanins are attractive model systems in protein crystallography [52]. The binding of Rh-containing fragments to the protein does not affect the main structural features of GpPC, which retains methylation of Asn72β and its ability to bind PCB. Twelve diRh binding sites were found on the protein structure, two for each (αβ) unit. The dimetallic fragments were found close to the side chains of Met30 of the β-chains and of Cys73 of the α-chains with similar occupancy factors for each diRh binding site, in line with the symmetric nature of the C-phycocyanin structure. Notably, to our knowledge, this represents the first unambiguous crystallographic observation of diRh compound binding to Met and Cys side chains. The results demonstrate the ability of these two residues to bind the diRh center at the axial site, and indicate that, at least in the case of Met, acetate ligands can be retained upon protein binding. Furthermore, data indicate that diRh binding to GpPC does not affect its thermal stability. These findings suggest that it is possible to design new diRh binding sites with the dirhodium center bound at the same time to His and Met, His and Cys, and Cys and Met side chains, as it occurs in the case of the His/His axial interaction observed by Jalilehvand et al. [42] and proposed for the Cys/Cys axial coordination by Garcia et al. [61]. Future studies will be devoted to studying the possible applications of diRh/GpPC crystals as a scaffold for heterogenous catalysis.

## Figures and Tables

**Table 1:** Data collection and refinement statistics.

PDB Deposition Code	9T05
Crystallization conditions	0.10–0.20 M magnesium chloride, 0.10 M Hepes, pH 6.5, and 9.0–10.0% (*w*/*v*) PEG 4000
Crystallization temperature (K)	293
Soaking temperature (K)	293
Data collection	
Data collection temperature (K)	100
Wavelength (Å)	0.9537
a (Å)/b (Å)/c (Å)	60.586/188.500/207.212
(αβ) per asymmetric unit	6 (12 chains: A, C, E, G, I, K for the α-chains and B, D, F, H, J, L for the β-chains)
Resolution range (Å)	188.5–2.17 (2.31–2.17)
Unique reflections	102,746 (5138)
Completeness (%)	94.2 (63.6)
Redundancy	12.9 (11.6)
† Rmerge (%)	0.340 (2.13)
Rpim	0.100 (0.656)
Average I/σ(I)	6.6 (1.4)
CC_1/2_	0.996 (0.564)
Anomalous completeness (%)	94.2 (65.5)
Anomalous redundancy	6.7 (6.0)
Refinement	
Resolution range (Å)	139.83–2.17
N. of reflections (working set)	97,759
N. of reflections (test set)	5000
R-factor/R-free (%)	22.7/26.3
N. of non-H atoms	16,492
Rh occupancy at Met30β binding site	0.50/0.50–B/0.50/0.50–D/0.50/0.50–F/0.50/0.50–H/0.50/0.50–J/0.60/0.60–L
Rh occupancy at Cys73α binding site	0.40/0.40–A/0.25/0.25–C/0.30/0.30–E/0.35/0.35–G/0.20/0.20–I/0.25/0.25–K
Average B-factors (Å^2^) All atoms	35.7
B-factors (Å^2^) of Rh atoms at Met30β binding site	44.0/48.8–B/47.7/49.1–D/58.6/59.7–F/43.5/49.6–H/33.8/34.1–J/24.0/26.7–L
B-factors (Å^2^) of Rh atoms at Cys73α binding site	37.2/62.5–A/47.8/52.8–C/48.4/61.8–E/40.7/65.7–G/30.9/57.0–I/54.0/58.5–K
R.m.s. deviations	
Bond lengths (Å)	0.008
Bond angles (°)	1.73
Ramachandran statistics (Coot analysis)	
Favored regions (%)/Outliers(%)	96.9/0.63

† Rmerge = ΣhΣi | I(h,i)-I(h)> | /ΣhΣi I(h,i), where I(h,i) is the intensity of the *i*th measurement of reflection. h and <I(h)> is the mean value of the intensity of reflection h.

**Table 2:** Geometric parameters of dirhodium centers bound to Met30β and Cys73α in the dirhodium/GpPC adduct.

Dirhodium/GpPC Adduct							
		Chain B	Chain D	Chain F	Chain H	Chain J	Chain L
Met30β	Rh—Rh ^(^Å)	2.38	2.38	2.39	2.38	2.41	2.36
	Rh—SD (Å)	2.47	2.39	2.51	2.51	2.44	2.44
	Rh—O_eq_ (Å)	2.04 ± 0.01	2.04 ± 0.01	2.05 ± 0.01	2.05 ± 0.01	2.05 ± 0.01	2.04 ± 0.01
	Rh—O_ax_^a^ (Å)	2.33	2.33	2.33	2.33	2.34	2.34
	SD—Rh—Rh (°)	170.5	172.8	177.1	172.1	173.3	176.6
	O_eq_—Rh—Rh ^b^ (°)	87.4 ± 4.8	87.1 ± 6.0	88.2 ± 0.7	87.8 ± 3.2	87.6 ± 2.4	86.5 ± 2.8
	O_eq_—Rh—SD (°)	92.2 ± 10.3	92.5 ± 7.0	91.6 ± 2.5	92.0 ± 9.6	92.2 ± 7.6	93.6 ± 2.3
	O_ax_^a^—Rh—Rh (°)	175.3	161.5	169.4	173.5	177.1	168.8
		**Chain A**	**Chain C**	**Chain E**	**Chain G**	**Chain I**	**Chain K**
Cys73α	Rh—Rh (Å)	2.41	2.40	2.40	2.40	2.38	2.39
	Rh—SG (Å)	2.13	2.47	2.26	2.14	2.08	2.47
	SG—Rh—Rh (°)	179.3	179.8	177.8	178.1	90.0	178.8

^a^ axial water ligand. ^b^ refers to the angle between O_eq_—Rh bound to the protein residue–Rh bound to the equatorial ligand.

## Data Availability

Data were deposited in the Protein Data Bank under the accession code 9T05.

## Supplementary Materials

The following supporting information can be downloaded at https://www.mdpi.com/article/10.3390/ijms262311492/s1.

## Abbreviations

bpy	Bipyridine
diRh	dirhodium
ESI-MS	Electrospray ionization mass spectrometry
EXAFS	extended X-ray absorption fine structure
GpPC	C-phycocyanin from *Galdiera phlegrea*
HEWL	Hen Egg White Lysozyme
HSA	Human serum albumin
MS	Mass spectrometry
PCB	phycocyanobilin
PDB	Protein Data Bank
