# Supplementary material for: Coordination of a Dirhodium(II) Center to Methionine and Cysteine Side Chains: Evidence from X-Ray Structure of the Adduct Formed by Dirhodium Tetraacetate with a C-Phycocyanin
Source: Int J Mol Sci. 2025 Nov 27;26(23):11492. doi: 10.3390/ijms262311492 (PMC12692400; doi:10.3390/ijms262311492)
Supplement: Supplementary file 1 [file ijms-26-11492-s001.zip › ijms-3968194-supplementary.pdf]

*Regular Article*

# Coordination of a Dirhodium(II) Center to Methionine and Cysteine Side Chains: Evidence from X-Ray Structure of the Adduct Formed by Dirhodium Tetraacetate with a C-Phycocyanin

**Giarita Ferraro, Paola Imbimbo, Romualdo Troisi, Daria Maria Monti and Antonello Merlino ***

Department of Chemical Sciences, University of Naples Federico II, Via Cintia, 80126 Napoli, Italy; giarita.ferraro@unina.it (G.F.); paola.imbimbo@unina.it (P.I.); romualdo.troisi@unina.it (R.T.); mdmonti@unina.it (D.M.M.)

* Correspondence: antonello.merlino@unina.it

**Table S1.** Rh binding sites in the structures of the Rh/HEWL and Rh/RNase A adducts formed upon reaction of dirhodium tetraacetate with the two proteins. The ligands at each Rh binding site are listed with their occupancy in parenthesis. * indicates atoms present in symmetry related molecules. d.c. = double conformation, Act=acetate

| Rh/ HEWL Adduct | | | | | | | | Rh/ RNase A Adduct | | | | | | | |
|---|---|---|---|---|---|---|---|---|---|---|---|---|---|---|---|
| **Crystallization conditions** | 0.01 M HEPES pH 7.5 and 2.0 M sodium formate | | 0.1 M sodium acetate, 20% ethylene glicol, 0.1 M sodium nitrate | | | | | 2.5 M NaCl 3.3 M HCOONa 0.1 M Acetate buffer | | | | | 22% PEG 4K 10 mM sodium citrate buffer pH 5.1 | | |
| **PDB code** | 9RUV | 7BE2 | 7BDZ | 7BE0 | 7BE1 | 7BEB | 7BEC | 8OQC | 8OQD | 8OQE | 8OQF | 8OQG | 6XVX | 6XW0 | 7QHR |
| **Binding sites** | **Metal fragments at binding sites** | | | | | | | | | | | | | | |
| **C-ter site** | Rh (0.50)<br>Rh*<br>(0.50)<br>Leu129<br>Leu129*<br>Lys13<br>Lys13* | Rh (0.45)<br>Rh *<br>(0.45)<br>$H_2O$ (0.5)<br>$H_2O$*<br>(0.5)<br>Leu129<br>Leu129*<br>Lys13<br>Lys13* | Rh (0.65)<br>Rh*(0.65)<br>Act (0.65)<br>Act (0.65)<br>$H_2O$ (0.65)<br>$H_2O$*(0.65)<br>Leu129<br>Leu129*<br>Lys13<br>Lys13* | Rh (0.65)<br>Rh*(0.65)<br>Act (0.65)<br>Act (0.65)<br>$H_2O$ (0.65)<br>$H_2O$*(0.65)<br>Leu129<br>Leu129*<br>Lys13<br>Lys13* | Rh (0.65)<br>Rh* (0.65)<br>Act (0.65)<br>Act (0.65)<br>$H_2O$ (0.65)<br>$H_2O$*(0.65)<br>Leu129<br>Leu129*<br>Lys13<br>Lys13* | Rh (0.7)<br>Rh* (0.7)<br>Act (0.7)<br>Act (0.7)<br>$H_2O$ (0.7)<br>$H_2O$* (0.7)<br>Leu129<br>Leu129*<br>Lys13<br>Lys13* | Rh (0.4)<br>Rh* (0.4)<br>Leu129<br>Leu129*<br>Lys13<br>Lys13* | - | - | - | - | - | - | - | - |
| **Lys33 site** | Rh (1)<br>$H_2O$ (1)<br>$H_2O$ (1)<br>$H_2O$ (1)<br>$H_2O$ (1)<br>Lys33 | Rh (0.20)<br>Act<br>(0.20)<br>Lys33 | - | - | - | - | - | - | - | - | - | - | - | - | - |
| **His15 site** | Rh (1)<br><br>$H_2O$ (1)<br>$H_2O$ (1)<br>His15<br>Arg14 | Rh (0.35)<br>$H_2O$<br>(0.35)<br>$H_2O$<br>(0.35)<br>His15<br>Arg14 | Rh (0.35)<br><br>Act (1)<br>$H_2O$ (0.35)<br>His15<br>Arg14 | Rh (0.5)<br>Act (1)<br>$H_2O$ (0.5)<br>His15<br>Arg14 | Rh (0.3)<br>Act (1)<br>His15<br>Arg14 (d.c.) | Rh (0.3)<br>Act (1)<br>$H_2O$ (0.3)<br>$H_2O$ (0.3)<br>His15 (1)<br>Arg14 (d.c) | Rh (0.25)<br>Act (1)<br>$H_2O$ (1)<br>His15<br>Arg14<br>(d.c.) | - | - | - | - | - | - | - | - |
| **Asp101 site** | Rh (0.60)<br>Rh (0.60)<br>Asp101 | Rh (0.40)<br>Rh (0.40)<br>$H_2O$<br>(0.40)<br>Asp101 | - | - | - | - | - | - | - | - | - | - | - | - | - |
| **Asn93 site** | Rh (0.50)<br>Rh (0.50)<br>Asn93<br>Lys96 | Rh (0.30)<br>Rh (0.30)<br>Asn93<br>Lys96<br>$H_2O$ (1) | - | - | - | - | - | - | - | - | - | - | - | - | - |

| | | | | | | | | | | | | | | | |
|---|---|---|---|---|---|---|---|---|---|---|---|---|---|---|---|
| **Asp18 site** | - | - | Rh (0.40)<br>Rh (0.40)<br>Asp18 | Rh (0.20)<br>Rh (0.20)<br>$H_2O$ (0.20)<br>Asp18 | - | - | - | - | - | - | - | - | - | - | - |
| **His119 site of molecule A** | - | - | - | - | - | - | - | Rh (0.50)<br>Rh (0.50)<br>Act (0.50)<br>Act (0.50)<br>Act (0.50)<br>$H_2O$ (0.50)<br>$H_2O$ (0.50)<br>Cl (0.50) | Rh (0.50)<br>Rh (0.50)<br>Act (0.50)<br>Act (0.50)<br>Act (0.50)<br>$H_2O$ (0.50)<br>$H_2O$ (0.50)<br>Cl (0.50) | Rh (0.55)<br>Rh (0.55)<br>OAc(0.55)<br>Act (0.55)<br>Act (0.55)<br>$H_2O$ (0.55)<br>$H_2O$ (0.55)<br>Cl (0.55) | Rh (0.35)<br>Rh (0.35)<br>Act (0.35)<br>$H_2O$ (0.35)<br>$H_2O$ (0.35) | Rh (0.35)<br>Rh (0.35)<br>$H_2O$ (0.35)<br>$H_2O$ (0.35)<br>$H_2O$ (0.35)<br>$H_2O$ (0.35) | Rh (0.60)<br>Rh (0.60)<br>Act (0.60)<br>Act (0.60)<br>Act (0.60)<br>Act (0.60)<br>$H_2O$ (0.60) | Rh (0.70)<br>Rh (0.70)<br>Act (0.70)<br>Act (0.70)<br>Act (0.70)<br>Act (0.70)<br>$H_2O$ (0.70) | Rh (0.80)<br>Rh (0.80)<br>Act (0.80)<br>Act (0.80)<br>Act (0.80)<br>$H_2O$ (0.80)<br>$H_2O$ (0.80)<br>$H_2O$ (0.80) |
| **His119 site of molecule B** | - | - | - | - | - | - | - | - | - | - | - | - | Rh (0.70)<br>Rh (0.70)<br>Act (0.70)<br>Act (0.70)<br>Act (0.70)<br>Act (0.70)<br>$H_2O$ (0.70) | Rh (0.70)<br>Rh (0.70)<br>Act (0.70)<br>Act (0.70)<br>Act (0.70)<br>Act (0.70)<br>$H_2O$ (0.70) | Rh (0.55)<br>Rh (0.55)<br>Act (0.55)<br>Act (0.55)<br>Act (0.55)<br>$H_2O$ (0.55)<br>$H_2O$ (0.55)<br>$H_2O$ (0.55) |
| **His105 site of molecule A** | - | - | - | - | - | - | - | Rh (0.78)<br>Rh (0.78)<br>Act (0.78)<br>Act (0.78)<br>Act (0.78)<br>Act (0.78)<br>Cl (0.75) | Rh (0.72)<br>Rh (0.72)<br>Act (0.72)<br>Act (0.72)<br>Act (0.72)<br>Act (0.72)<br>Cl (0.70) | Rh (0.78)<br>Rh (0.78)<br>Act (0.78)<br>Act (0.78)<br>Act (0.78)<br>Act (0.78)<br>Cl (0.78) | Rh (0.75)<br>Rh (0.75)<br>Act (0.75)<br>Act (0.75)<br>Act (0.75)<br>Act (0.75)<br>Cl (0.75) | Rh (0.80)<br>Rh (0.80)<br>Act (0.80)<br>Act (0.80)<br>Act (0.80)<br>Act (0.80)<br>Cl (0.80 | Rh (0.60)<br>Rh (0.60)<br>Act (0.60)<br>Act (0.60)<br>Act (0.60)<br>Act (0.60)<br>$H_2O$ (0.60) | Rh (0.60)<br>Rh (0.60)<br>Act (0.60)<br>Act (0.60)<br>Act (0.60)<br>Act (0.60) | Rh (0.50/0.50) (d.c.)<br>Rh (0.50/0.50) (d.c.)<br>$H_2O$ (0.50/0.50) (d.c.)<br>$H_2O$ (0.50/0.50) (d.c.)<br>Act (0.50)<br>$H_2O$ (0.50) |

| | | | | | | | | | | | | | | | |
|---|---|---|---|---|---|---|---|---|---|---|---|---|---|---|---|
| **His105 site of molecule B** | - | - | - | - | - | - | - | - | - | - | - | - | Rh (0.60)<br>Rh (0.60)<br>Act (0.60)<br>Act (0.60)<br>Act (0.60)<br>Act (0.60)<br>$H_2O$ (0.60) | Rh (0.60)<br>Rh (0.60)<br>$H_2O$ (0.60)<br>$H_2O$ (0.60)<br>$H_2O$ (0.60) | Rh (0.70)<br>Rh (0.70)<br>Act (0.70)<br>$H_2O$ (0.70)<br>$H_2O$ (0.70)<br>$H_2O$ (0.70)<br>$H_2O$ (0.70)<br>$H_2O$ (0.70) |

**Table S2.** Summary of the Rh-containing fragments found in the five structures of Rh/protein adducts obtained upon reaction of *cis*-[$Rh_2(\mu\text{-}O_2CCF_3)_2(\mu\text{-}O_2CCH_3)_2$] with RNase A or HEWL. The Rh ligands identified in each binding site are described with occupancy values in parentheses. d.c. and ax refer to double conformations and axial ligands, respectively; tfa=trifluoroacetic acid, act=acetic acid)

| **Rh/ HEWL Adduct** | | **Rh/ RNase A Adduct** | | | |
|---|---|---|---|---|---|
| **Crystallization conditions** | 0.01 M HEPES pH 7.5 2.00 M sodium formate | 22 % PEG 4K 0.01 M sodium citrate pH 5.1 | | | |
| **PDB code** | 7QQ1 | 7QPW | 7QQ0 | 7QPY | 7QPZ |
| **Binding sites** | **Metal fragments at binding sites** | | | | |
| **His15 site** | Rh (0.50)<br>Rh (0.50) | - | - | - | - |
| **Asp101 site** | Rh (0.30)<br>$H_2O$ (0.30) | - | - | - | - |
| **His119 site of molecule A** | - | Rh (0.55)<br>Rh (0.55)<br>Act (0.55)<br>tfa (0.55)<br>$H_2O$ (0.55)<br>$H_2O$ (0.55)<br>$H_2O_{ax}$ (0.55) | Rh (0.40)<br>Rh (0.40)<br>Act (0.40)<br>$H_2O$ (0.40)<br>$H_2O$ (0.40)<br>$H_2O$ (0.40)<br>$H_2O$ (0.40)<br>$H_2O$ (0.40)<br>$H_2O$ (0.40) | Rh (0.80)<br>Rh (0.80)<br>Act (0.80)<br>tfa (0.80)<br>$H_2O$ (0.80)<br>$H_2O$ (0.80)<br>$H_2O_{ax}$ (0.80) | Rh (0.75)<br>Rh (0.75)<br>Act (0.75)<br>tfa (0.75)<br>$H_2O$ (0.75)<br>$H_2O$ (0.75)<br>$H_2O_{ax}$ (0.75) |
| **His119 site of molecule B** | - | Rh (0.20) (d.c.)<br>Rh (0.20) (d.c.)<br>Act (0.20) (d.c.)<br>Act (0.20) (d.c.)<br>$H_2O$ (0.20) (d.c.)<br>$H_2O$ (0.20) (d.c.)<br>$H_2O$ (0.20) (d.c.)<br>$H_2O$ (0.20) (d.c.)<br>$H_2O_{ax}$ (0.40) | Rh (0.40)<br>Rh (0.40)<br>Act (0.40)<br>$H_2O$ (0.40)<br>$H_2O$ (0.40)<br>$H_2O$ (0.40)<br>$H_2O$ (0.40)<br>$H_2O$ (0.40)<br>$H_2O$ (0.40)<br>$H_2O_{ax}$ (0.40) | Rh (0.30/0.30) (d.c.)<br>Rh (0.30/0.30) (d.c.)<br>Act (0.30/0.30) (d.c.)<br>Act (0.30/0.30) (d.c.)<br>$H_2O$ (0.30/0.30) (d.c.)<br>$H_2O$ (0.30/0.30) (d.c.)<br>$H_2O$ (0.30/0.30) (d.c.)<br>$H_2O$ (0.30/0.30) (d.c.)<br>$H_2O_{ax}$ (0.60) | Rh (0.30/0.30) (d.c.)<br>Rh (0.30/0.30) (d.c.)<br>Act (0.30/0.30) (d.c.)<br>Act (0.30/0.30) (d.c.)<br>$H_2O$ (0.30/0.30) (d.c.)<br>$H_2O$ (0.30/0.30) (d.c.)<br>$H_2O$ (0.30/0.30) (d.c.)<br>$H_2O$ (0.30/0.30) (d.c.)<br>$H_2O_{ax}$ (0.60) |
| **His105 site of molecule A** | - | Rh (0.40)<br>Rh (0.40)<br>Act (0.40)<br>$H_2O$ (0.40)<br>$H_2O$ (0.40)<br>$H_2O$ (0.40)<br>$H_2O$ (0.40)<br>$H_2O$ (0.40) | Rh (0.40)<br>Rh (0.40)<br>$H_2O$ (0.40) | Rh (0.70)<br>Rh (0.70)<br>Act (0.70)<br>$H_2O$ (0.70)<br>$H_2O$ (0.70)<br>$H_2O$ (0.70)<br>$H_2O$ (0.70)<br>$H_2O$ (0.70) | Rh (0.55)<br>Rh (0.55)<br>Act (0.55)<br>Act (0.55)<br>$H_2O$ (0.55)<br>$H_2O$ (0.55)<br>$H_2O$ (0.55)<br>$H_2O$ (0.55) |

| | | | | | |
|---|---|---|---|---|---|
| | | $H_2O$ (0.40)<br>$H_2O_{ax}$ (0.20) (d.c.) | | $H_2O$ (0.70)<br>$H_2O_{ax}$ (0.70) | |
| **His105 site of molecule B** | - | - | - | Rh (0.40)<br>Rh (0.40)<br>$H_2O$ (0.40)<br>$H_2O$ (0.40)<br>$H_2O$ (0.40)<br>$H_2O$ (0.40)<br>$H_2O$ (0.40)<br>$H_2O$ (0.40)<br>$H_2O$ (0.40)<br>$H_2O$ (0.40) | - |

**Table S3.** Rh-containing fragments and ligands bound in the three structures of Rh/protein adducts obtained upon reaction of $[Rh_2(\mu\text{-}O_2CCF_3)_3(\mu\text{-}O_2CCH_3)_2]$ with RNase A and HEWL. Values in parenthesis indicate the occupancy of the metals and ligands. d.c. = double conformation, act=acetate

| Rh/ HEWL Adduct | | Rh/ RNase A Adduct | |
|---|---|---|---|
| **Crystallization conditions** | 0.01 M HEPES pH 7.5 2.00 M sodium formate | 22 % PEG 4K 0.01 M sodium citrate pH 5.1 | |
| **PDB code** | 7Z6J | 7Z6D | 7Z6G |
| **Binding sites** | **Metal fragments at binding sites** | | |
| **His15 site** | Rh (0.25)<br>Rh (0.25) | - | - |
| **Lys33 site** | Rh (0.30)<br>$H_2O$ (0.30) | - | - |
| **Asp101 site** | Rh (0.25)<br>$H_2O$ (0.25) | - | - |
| **Leu129 site** | Rh (0.30)<br>Rh*(0.30) | - | - |
| **His119 site of molecule A** | - | Rh (0.40)<br>Rh (0.40) | Rh (0.55)<br>Rh (0.55) |
| **His119 site of molecule B** | - | Rh (0.20) (d.c.)<br>Rh (0.20) (d.c.)<br>Rh (0.40) (d.c.)<br>Rh (0.40) (d.c.)<br>$H_2O$ (0.20) (d.c.)<br>$H_2O$ (0.40) (d.c.)<br>$H_2O$ (0.40) (d.c.)<br>$H_2O$ (0.40) (d.c.)<br>$H_2O$ (0.40) (d.c.) | Rh (0.55) (d.c.)<br>Rh (0.55) (d.c.)<br>Rh (0.30) (d.c.)<br>Rh (0.30) (d.c.)<br>$H_2O$ (0.55) (d.c.)<br>$H_2O$ (0.55) (d.c.)<br>$H_2O$ (0.55) (d.c.)<br>$H_2O$ (0.55) (d.c.)<br>$H_2O$ (0.30) (d.c.) |
| **His105 site of molecule A** | - | Rh (0.50)<br>Rh (0.50)<br>Act (0.50)<br>$H_2O$ (0.50)<br>$H_2O$ (0.50)<br>$H_2O$ (0.50) | Rh (0.70)<br>Rh (0.70)<br>Act (0.70)<br>$H_2O$ (0.70)<br>$H_2O$ (0.70)<br>$H_2O$ (0.70) |
| **His105 site of molecule B** | - | - | - |
